# Monkeypox: Origin, Transmission, Clinical Manifestations, Prevention, and Therapeutic Options

**DOI:** 10.1155/ipid/2522741

**Published:** 2025-02-02

**Authors:** Sajal Kumar Halder, Arafin Sultana, Mahbubul Kabir Himel, Aparna Shil

**Affiliations:** ^1^Department of Biochemistry and Molecular Biology, Jahangirnagar University, Savar, Dhaka 1342, Bangladesh; ^2^Division of Computational Biology, Padma Bioresearch, Savar, Dhaka, Bangladesh; ^3^Department of Botany, Jahangirnagar University, Savar, Dhaka 1342, Bangladesh

**Keywords:** antiviral therapy, global public health, monkeypox, transmissible disease, vaccine development

## Abstract

Monkeypox is a rapidly spreading transmissible disease induced by the monkeypox virus (MPXV), a major public health problem worldwide. The origin of monkeypox might be tracked to the continent of Africa, where it first afflicted primate species prior to spreading to the world. Severe health issues for the public have been raised as a result of the disease's current breakouts in nonendemic areas and its subsequent dissemination to several nations throughout the globe. Monkeypox spreads by having contact with infected creatures or people, as well as respiratory droplets and contaminated things. Symptoms of monkeypox in young children and adults are different. While the symptoms are similar to smallpox, monkeypox has a reduced mortality rate. Proper diagnosis, suitable care, and focused preventative efforts all depend on becoming cognizant of those distinctions. Numerous promising therapeutic approaches have been recently investigated. Antiviral drugs such as tecovirimat, cidofovir, and brincidofovir, which were initially developed to treat smallpox, were found to have been effective in treating MPXV cases. Moreover, vaccinations continue to be an important preventative step. The purpose of this article is to offer the most recent and thorough information available on monkeypox, including its possible causes, modes of transfer, and potential treatments. By identifying the distinct forms of monkeypox and exploring potential treatment options, this work contributes to the ongoing battle against MPXVs and the management of this novel viral disease. To stop the propagation of monkeypox, greater research and communication are needed to provide stronger treatments and effective vaccinations.

## 1. Introduction

**Figure 1:**
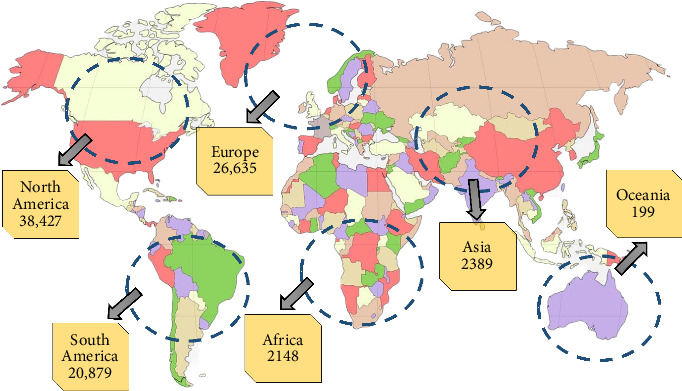
A worldwide map depicting the spread of monkeypox (Mpox) cases depending on confirmed cases as of December 01, 2023.

The monkeypox virus (MPXV) is a DNA virus with double strands causing a transmissible disease known as monkeypox (Mpox) in humans and other mammals [1, 2]. Over 92,167 confirmed and suspected cases of Mpox have been documented in 117 different countries, demonstrating the global prevalence of this disease (as of December 01, 2023) (Figure 1) [3]. While scientific understanding of Mpox has increased in recent years, comprehensive and up-to-date information about the illness remains limited [4]. Therefore, this review aims to shed light on Mpox, covering its prevalence, transmission clinical manifestations, prevention measures, vaccination strategies, and potential therapeutic targets and potential treatment options.

The spread of human Mpox across several nations resulted in serious public health downfall around the globe. Outbreaks in *Macaca fascicularis* monkeys in Copenhagen, Denmark, in 1959 led to the discovery of the causal agent, MPXV [5]. This disease subsequently spread to monkeys kept in zoos and research facilities [6]. Mpox is a zoonotic disease since it initially appeared in a person in the Democratic Republic of the Congo around 1970 [7]. Following that point, there have been isolated outbreaks of Mpox and reports of human-to-human transfer throughout Western and Central Africa [8–10].

The rise in Mpox epidemics over the last three decades is likely due to a combination of numerous factors [11]. The weakened immunity caused by the discontinuation of smallpox vaccination is a key element, as smallpox vaccination provides about 85% immunity to the virus [12]. Furthermore, the danger of contact with MPXV repository species has risen due to increasing people volume, the simplicity of movement, and environmental alterations like deforestation [13–15]. Widespread consumption of possible reservoir species has occurred in places where impoverishment and societal emergencies, such as armed conflicts, are prevalent [13]. These factors collectively underscore the global relevance of Mpox as an emerging zoonosis [16].

Symptomatically, Mpox is very comparable to smallpox; however, it has a significantly reduced fatality rate [17]. World Health Organization (WHO) announced an international health crisis due to a sharp rise in the cases of Mpox throughout the entire world [18]. Prior smallpox immunization has shown effective in averting Mpox, with vaccinated persons having a far lower risk of contracting the viral infection [19]. However, a study during the current outbreak revealed that only a small percentage of infected individuals had received prior smallpox vaccination [17, 20]. Interestingly, this outbreak has also disproportionately affected men who have sex with men (MSM). The positive MPXVs are identified by PCR, serological detection (antibodies) against the MPXV, or histological examination [21–23].

Although there is currently no FDA-approved therapy for Mpox infection, several antiviral drugs that are often utilized for treating smallpox (such as tecovirimat, cidofovir, and brincidofovir) might prove helpful for infected patients. The demand for novel oral drugs is growing over time, and funding research into disease mechanisms and drug discovery can help meet this need. This research gap is required to be minimized by interconnecting the research among diverse groups around the world [24]. Researchers are looking at MPXV genetic modifications to see whether they have played a part in the recent epidemic [25].

The purpose of this paper is to offer a current and thorough description of Mpox, including its epidemiology, etiology, pathogenesis, clinical characteristics, laboratory results, complications, and preventative measures such as vaccine development and available treatment options. We will also review the host's immune systems and key information discrepancies, as well as investigate the distinct epidemiological and clinical features found throughout the latest epidemic.

## 2. Origin and Transmission of Mpox

### 2.1. Origin of Mpox

In an epidemic of vesicular illness in Copenhagen in 1958, the MPXV was originally identified in captive cynomolgus monkeys. As a result, the disease is known as “monkeypox” and given recognition as a specific member of the group [5]. However, the name is confusing because the virus's biggest animal hosts have been discovered in rodents, such as squirrels and giant pouched rats [26]. Scientists believe that MPV originated from a companion monkey. Unfortunately, it is unclear whether MPV originated from a recent (nasopharyngeal colonization) or distant (latent carrier of virus in tissues) infection. The second possibility is supported by the discovery of a virus that is similar to MPV in the kidneys of otherwise healthy monkeys [27, 28]. The prototype virus was rectangular in shape and size (200 by 250 nm), similar to other known smallpox viruses. Following that, Marennikova et al. evaluated the attributes of five MPV variants; four of the five MPV variants tested had biological traits that were similar to vaccinia viruses [29]. Because the vaccinia vaccine protected against human smallpox, people who came in contact with infected animals were unaffected. And as long as smallpox immunization was maintained, the disease was not perceived as a threat.

A 9-month-old baby in the Equateur region of the Democratic Republic of the Congo had a smallpox-like clinical condition in 1970, even though smallpox virus transmission had been known to have ceased in the area. Subsequently, five more patients from Liberia and Sierra Leone were confirmed in the same year [27]. Initially, each case was diagnosed as smallpox based on clinical signs of infection in all six patients who were unvaccinated. Due to the antigenic similarities between the two viruses, the smallpox vaccine, which contains the vaccinia virus, offers cross-protection against MPXVs. By producing an immune response that is effective against MPXVs, this cross-protection dramatically lowers the incidence and severity of Mpox in those who have received vaccinations. The discontinuation of smallpox vaccination programs has resulted in a population with reduced immunity, which has contributed to the resurgence of Mpox [30, 31]. However, many benchmark laboratories thoroughly examined the substances recovered from these individuals, and all have been recognized as MPV. It spurred concern about whether Mpox could eventually overtake smallpox in unvaccinated populations. Mpox did not kill any of the patients. Furthermore, none of the patients' family members contracted the condition. A record of 59 cases of human Mpox was discovered in Cameroon, Cote d'lvoire, Liberia, Nigeria, Sierra Leone, and the Democratic Republic of the Congo between 1970 and 1980.

In 1981, national authorities in the Democratic Republic of the Congo began an intensive active surveillance program for human Mpox with the help of WHO [32]. From 1992, there were no further cases of human Mpox notified to WHO until 1996, when Medecins Sans Frontières (MSF) notified the WHO of a probable Mpox outbreak in the Katako Kombe subregion of Kasai Oriental [32].

Mpox outbreaks have been documented in 11 African nations over the year, with the Democratic Republic of the Congo reporting the most cases [33]. Between May 4 and 25 , 221 verified and 86 suspected cases of Mpox were registered from 23 European nations, as well as Argentina, Australia, Canada, the United Arab Emirates, and the United States [34]. In just a few weeks, such a significant number of cases have never been reported from so many countries outside of Africa. This could be linked to a decrease in population immunity to smallpox over time. Children who had not received smallpox immunization have historically had the greatest rates of disease [35].

### 2.2. Transmission of Mpox

Although the exact animal host reservoir for Mpox is obscure, rodents are thought to play a role in Mpox transmission to humans. Animals have been shown to transmit aerosols, which could explain a nosocomial outbreak of Mpox [36]. Indirect or direct contact with live or dead animals, on the other hand, is thought to be the cause of human Mpox outbreaks [37, 38]. Mpox is transmitted in two ways: primary transmission and secondary transmission [39].

**Figure 2:**
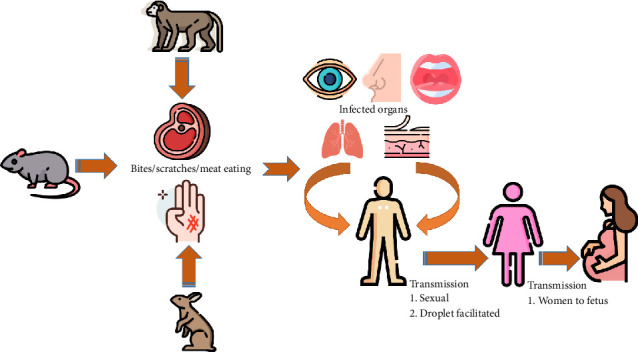
The modes of transmission of the monkeypox (Mpox) virus to humans.

The MPXV can be transmitted from animals to humans by bites or scratches, the use of items derived from infected animals, or direct contact with body fluids or sores on an infected person or materials that have come in contact with body fluids or wounds [39]. People are forced to hunt mammals for protein-rich food due to low socioeconomic status, increasing their exposure to wild rodents, which may carry Mpox [40]. It is hypothesized that the virus enters the body through broken skin, the respiratory tract, or mucous membranes (eyes, nose, or mouth) [40]. Direct, aerosol, and fomite are the three modes of transmission of the MPXV from animal to human [39, 40]. The majority of researchers' recommendations for probable MPXV transmission pathways included direct transmission. Aerosol and fomite transmission have also been postulated as possible mechanisms for MPXVs to spread from animals to humans [40]. Orthopoxvirus antigens were found in abundance in the surface epithelial cells of lesions in the conjunctiva and tongue, but in less amounts in surrounding macrophages, fibroblasts, and connective tissues. In bronchial epithelial cells, macrophages, and fibroblasts, viral antigens were numerous; virus isolation and electron microscopy revealed active viral replication in the lungs and tongue. These data imply that both respiratory and direct mucocutaneous exposures may play a role in MPXV transmission between rats and humans (Figure 2) [39].

Secondary human-to-human transfer is a rather typical occurrence [32]. In secondary transmission, whenever a person comes into touch with the virus from an infected individual, or contaminated objects, the infection can spread. Mpox can be spread by close personal contact. Also, respiratory secretions can potentially spread during prolonged face-to-face contact. To present, there are no data that human-to-human transmission can keep MPXVs alive in the human community (Figure 2) [41, 42]. They are more likely to be exposed to the virus through both penetrating and nonpenetrating sexual contact. Dr. Nicola Low and her colleagues reported epidemiological, clinical, and virological findings, showing an increase in the percentage of infection with MSM network in infectious lesions compared to respiratory droplets or sperm [43].

### 2.3. Mpox in Children

Mpox can infect people of any age group; cases have been reported ranging from 3-month-old newborns to 69-year-olds. According to WHO data, people under the age of 15 account for 92.5% of all Mpox cases, with half of them being under the age of five [44]. Up to 6 months, maternal immunization may partially protect the infant, and there is a lower risk of exposure to a case of Mpox at that age. One study examined at 214 cases and found that the majority of Mpox patients were children under the age of 15 [45]. According to another study, children aged 5 to 9 years had the highest incidence of MPXV-specific antibodies (13.1 per 1000) [46]. Mpox is a greater threat to children, owing to the lack of vaccination at a young age. As a result, they are more susceptible to the disease [47]. Some symptoms of Mpox, particularly in children, are similar to those of chicken pox, such as rashes, fever, and pains [47]. The virus can also be transferred “through delivery and early physical contact” to a fetus or newborn kid. Also, children have greater case fatality rates than adults [48].

Mpox symptoms include fever and rash in children. Toxemia and viremia cause the initial fever. Children's headaches and backaches are not particularly bothersome. Vomiting was reported by a large number of people, and it subsided when the rash appeared. It recurred in some children during the pustular stage and then faded as scars formed [48].

Mpox is now without a recognized or effective treatment, and the majority of victims recover and survive without it. Children with MPXVs were administered with smallpox vaccine in addition to supportive treatment. Supportive treatment includes managing symptoms such as fever, pain, and dehydration. This calls for the use of fluids to stay hydrated, analgesics, and antipyretics [49].

### 2.4. Mpox in Elderly

The clinical signs of Mpox in adults are poorly understood, and clinical laboratory findings are unexplained [45]. Despite the fact that Mpox is more severe in children, it can infect adults of any age. Since the majority of adults have had the smallpox vaccine, the number is quite modest. However, an outbreak of Mpox in the Bangassou district in 2015 revealed that adults were the ones who were most impacted by the disease (8 out of 12), where the mean age was 25 years with extremes at 15 months and 41 years [50]. Adults in an endemic population would have previously been exposed to the virus at some point in their lives, and because the only two conceivable outcomes of infection were mortality or survivability with life-long immunity, the virus could only infect and spread to those who had never been infected before [50]. Adults over the age of 18 have been assessed to be at high risk of contracting smallpox or Mpox, according to the U.S. Food and Drug Administration. Adult actions such as hunting and carcass preparation may play a role in MPXV transmission [51].

In the United States in 2003, one study looked at clinical and laboratory aspects of human Mpox infection in adults and children to see whether there were any risk factors for acute infection and hospitalization. When the results of pediatric and adult patients were evaluated, it was discovered that adult patients were much less likely to be admitted to the intensive care unit [52]. However, the massive outbreak of human Mpox in West Africa in 2017 predominantly affected adults. In Kikwit, Zaire, a fatal Mpox outbreak revealed that the victims were largely unprotected young children, as most adults had been vaccinated [53].

The symptoms of Mpox in adults are similar to those in youngsters. Fever, headaches, muscle aches, and tiredness are the initial symptoms. The critical difference between smallpox and Mpox symptoms is that Mpox causes swollen lymph nodes (lymphadenopathy), but smallpox does not. Mpox can also be caught by older people who have been vaccinated, but the symptoms are relatively mild [48, 49].

### 2.5. Therapeutic Strategies for Mpox

There are currently no approved therapeutic options for human Mpox [54]. Fortunately, antivirals designed for the treatment of smallpox patients could be helpful. We have categorized the antiviral agents into small drug-like molecules, peptides, interferons, nanoparticles, and RNA interference (RNAi) groups (Table 1).

## 3. Small Drug-Like Molecules

### 3.1. Tecovirimat (ST-246)

Tecovirimat, commonly marketed as Tpoxx, is an antiviral drug that fights against orthopoxviruses including Mpox and smallpox [71]. It was discovered from the various dataset of 356,240 ligands [72]. In an S9 in vitro experiment where the metabolic activity is measured, ST-246 shows a steady half-life (t1/2) of > 200 min and a 50% effective concentration of 25 nM [73]. ST-246 works by inhibiting the development of egress-competent from orthopoxviruses [63]. ST-246 acts on the p37 protein of the poxvirus, which is evolutionarily conserved for all orthopoxviruses [65]. It prevents the viral components from escaping affected cells. The protein is essential for the synthesis of viral extracellular forms, which is necessary for the formation of viral external shapes. ST-246 is bioavailable when administered Tpoxx [63].

ST-246 is insoluble in water and gastric liquids, although it was shown to be highly permeable in Caco-2 tests, indicating excellent gastrointestinal absorption. ST-246 is mostly excreted in the feces, with a minor quantity excreted in the urine. When consumed with meals, the bioavailability of ST-246 is enhanced [63]. ST-246 does not produce bone marrow poisoning or genetic defects in the mouse micronucleus experiment, and it is also not genotoxic in microbial and animal genotoxicity experiments. There was no indication of infertility, fetal resorptions, fetal abnormalities, or poisoning in rabbits and mice [63]. On July 13, 2018, the U.S. Food and Drug Administration authorized tecovirimat as the therapeutic agent of smallpox [8]. Health Canada approved this drug in December 2021 [74].

### 3.2. Cidofovir

Cidofovir is a nucleotide derivative having antimicrobial efficacy toward cytomegalovirus (CMV) and herpesviruses [75]. It is highly effective in all orthopoxviruses studied, notably Mpox, vaccinia, and variola viruses in cell cultures [76]. Cidofovir generally inhibits DNA polymerase [76]. The active form of cidofovir (cidofovir diphosphate) blocks the activity of DNA polymerase. This suppression is due to cidofovir diphosphate competing with deoxycytidine triphosphate (dCTP) as an alternate compound [58]. The integration of cidofovir into the developing viral DNA chain decreases the occurrence of DNA production [77]. Cidofovir shows a limited oral bioavailability (less than 5%); thus, it is given as an injection [78]. About 90% of a cidofovir dosage taken is excreted intact in the urine [79]. Side effects linked with intravenous cidofovir cause therapeutic withdrawal (∼20%–25% cases), commonly due to nephrotoxicity. Fever (6.2%), skin rash or pruritus (6.2%), nausea and vomiting (9.1%), hematological abnormalities (9.7%), uveitis/iritis (10.1%), and nephrotoxicity (22.4%) are the common side effects [79].

### 3.3. Brincidofovir

Brincidofovir is an antiviral agent administered orally for human smallpox diseases. It is licensed and sold in European countries and the United States as an antiviral medication [80]. In June 2021, the FDA authorized brincidofovir, marketed as Tembexa by Chimerix, for treating smallpox [61]. Recently, numerous health organizations have endorsed brincidofovir as an effective treatment for Mpox [81]. The lipid portion enhances therapeutic distribution to recipient cells and lowers nephrotoxicity [67, 82]. Brincidofovir acts as a prodrug enabling oral dose instead of intravenous injection. It enhances the bioavailability of cidofovir [61, 83]. Because cidofovir has a wide antiviral effect against various viral DNA, brincidofovir can act as a therapeutic agent for Epstein–Barr virus (EBV), adenoviruses (AdV), BK virus (BKV), and cytomegalovirus (CMV) [67]. The lipid moiety resembles native molecules, lysophosphatidylcholine, allowing the compound to penetrate invading pathogens via native lipid absorption routes [45, 47]. After absorption, the lipid part is broken to produce cidofovir, which is subsequently phosphorylated to yield cidofovir diphosphate, the active antiviral agent [59]. Therefore, the diphosphate can integrate into the increasing viral DNA target, slowing the pace of viral DNA formation [59]. Sphingomyelinase subsequently hydrolyzes and metabolizes the drug [80]. BCV-related nephrotoxicity is uncommon, and no stringent dosage changes are necessary for the presence of renal impairment [59, 80].

### 3.4. Nucleoside/Nucleotide Analogs

Various nucleoside and nucleotide derivatives have been reported to have effective antiviral activity for poxviruses (Mpox, variola, cowpox, vaccinia) [84]. Based on human trials, these analogs should be further studied for their prophylactic and therapeutic efficacy in the treatment of Mpox diseases [85]. Clercq et al. suggested (S)-1-(3-hydroxy-2-phosphonylmethoxypropyl) cytosine [(S)-HPMPC, cidofovir], (S)-9-(3-hydroxy-2-phosphonylmethoxypropyl)-2,6-diaminopurine [(S)-HPMPDAP], and (S)-6-(3-hydroxy-2-phosphonylmethoxypropyl) oxy-2,4-diaminopyrimidine [(S)-HPMPO-DAPy] as the potential antipoxvirus drug. Similarly, nucleoside analog, 8-methyladenosine and 2-amino-7-[(1,3-dihydroxy-2-propoxy) methyl] purine (S2242), have shown antiviral activity toward poxviruses [84]. The analogs have been shown to be successful in preventing poxvirus replication. They target the following viral proteins: (1) thymidylate synthase, which converts dUMP to dTMP, a critical intermediary in the creation of dTTP; (2) IMP dehydrogenase, responsible for converting IMP to XMP, crucial in the biogenesis of GTP and dGTP; (3) CTP synthetase, which is responsible for the biogenesis of CTP, namely, the transition of UTP to CTP; (4) OMP decarboxylase, that changes OMP to UMP, a vital step in the creation of pyrimidine mononucleotides; and (5) SAH hydrolase, which is responsible for the breakdown of S-adenosylhomocysteine (SAH) [84]. However, the underlying mechanisms of antiviral activity are still unknown [85].

Smee et al. discovered that N-methanocarbathymidine [(N)-MCT] might suppress orthopoxvirus replication in tissue culture and mice [64]. According to toxicological studies in mice, the toxicity of (N)-MCT proved to be less than cidofovir and acceptable at the highest concentration of 1000 mg/kg/day [64]. The molecule may interfere with viral DNA production by inhibiting orthopoxvirus and herpesvirus DNA polymerases. Alternatively, the monophosphate variant of (N)-MCT may block thymidylate synthetase interfering with orthopoxvirus DNA synthesis [86]. Kern et al. revealed that 1-(2-deoxy-4-thio-d-ribofuranosyl)-5-iodouracil (4′-thioIDU) had antiviral activity against orthopoxvirus infections resistant to cidofovir or tecovirimat [87]. 4′-ThioIDU has anticowpox virus action by inhibiting the viral thymidine kinase [66]. KAY-2-41 (1′-carbon-substituted 4′-thiothymidine derivative) showed antiviral activity against the vaccinia, cowpox, and camelpox viruses, according to Duraffour et al. [88]. It had better potency than cidofovir but was less effective than CDV or tecovirimat [67]. In mice, viral replication was eliminated following the treatment of KAY-2-41 (50 mg/kg) to the principal target tissues (lungs, liver, spleen, and kidneys) [88].

Oleg et al. found NIOCH-14 (derivative of tricyclodicarboxylic acid) as a potential antismallpox through in vitro and in vivo analysis [69]. However, NIOCH-14 did not block the replication of coxsackievirus A7, West Nile virus, adenovirus serotype 5, herpes simplex virus type 2, or coxsackievirus A7 [69]. NIOCH-14 suppressed virus quantity in critical organs (trachea, nose, and lungs) in assays utilizing 10 ID50 of Ectromelia virus (ECTV) and ICR mice. Antibody levels are high in the ICR mice of Ectromelia virus ECTV, VARV (variola virus), and MPXVs [67]. Connor et al. have noted CMLDBU6128 as a promising antiviral drug in vitro against orthopoxviruses [89]. CMLDBU6128 was found to be a precise inhibitor of poxvirus, with efficacy toward cowpox, vaccinia, and Mpox and no efficacy toward RNA viruses, adenoviruses, or HSVs [70]. The compound's IC50 was in the single micromolar range, indicating that it possesses decreased ability to block initial phases of vaccinia gene expression but strong suppression of late gene expression and multiplication [70]. CMLDBU6128 inhibits the transcription of vaccinia virus, disrupting J6R function preferentially [89].

## 4. Vaccines

Smallpox immunization has been found to be protective against Mpox in the past [90]. According to statistics across Africa, the smallpox vaccine is at least 85% efficient in preventing Mpox [30]. The vaccines for smallpox can protect against Mpox infections because it is a closely related variola virus that causes smallpox [30]. However, immunity to the smallpox vaccine will be reduced in the elderly below the range of 40 or 50 years [90]. Furthermore, following vaccination, immunity may have diminished over years [30, 31, 68].

### 4.1. Vaccines of the First Generation

Locally developed vaccines achieved domestic popularity via preliminary experiments and became first-generation vaccinations. These vaccines were not clonal and pure, and they were sequentially produced on farm animals, commonly calves or sheep, implying that microbe infection was common [91]. During the smallpox control and elimination period, four major vaccines were found: EM-63, which was revolutionary in the Soviet Union; Lister, which was used in the United Kingdom; Temple of Heaven, which was available in China; and Dryvax^TM^, which was used in the United States [19, 92].

### 4.2. New Vaccines

#### 4.2.1. Imvamune

The FDA has approved Imvamune (also known as MVA-BN, JYNNEOS, Imvanex), a live attenuated vaccine, for the treatment of Mpox and smallpox [92, 93]. Bavarian Nordic's Imvamune is a customized vaccinia virus Ankara produced from a replication-competent viral vector vaccinia Ankara [94]. It has passed six cycles of plaque filtration and is maintained in serum-deficient environments following > 570 uninterrupted rounds in the embryo of chicken fibroblasts [95]. The replication competence in human cell lines has been lost because of its high degree of attenuation. The data from completed and ongoing clinical trials covering 7600 people, including HIV-positive and atopic dermatitis patients, revealed that most of the events were mild to moderate [95]. The vaccination was tolerated in all the groups without any significant variance [95]. The researchers noted one unverified instance of potential acute pericarditis in the Phase 3 clinical trial was caused by the Imvamune. Moreover, no documented incidences of myopericarditis or other heart inflammation were reported in the clinical trial. Imvamune is administered through a dermal injection, usually in the upper part of the arms [95]. People immunized without smallpox must take dual doses of 0.5 mL Imvamune, the second one after 28 days of the first dose [96]. Imvanex includes vaccinia Ankara, a customized version of the vaccinia that does not induce infection and duplicate in the human body [93]. Because of the similarities, antibodies developed against this virus are predicted to defend themselves from smallpox [95]. Vaccines incorporating the vaccinia virus have proven successful in the smallpox eradication program. Weariness, headache, nausea, and site of injection (itching, pain, and redness) responses are among the most prevalent Imvamune adverse effects [96]. Imvamune should not be provided to people who are sensitized to the active or inactive element [96].

#### 4.2.2. ACAM2000

In August 2007, the U.S. Food and Drug Administration approved a novel vaccine ACAM2000 to prevent smallpox [97]. Sanofi Pasteur Biologics, LLC manufactures ACAM2000, a live vaccinia virus smallpox vaccine developed from formerly authorized Dryvax and synthesized in Vero cells [95]. In Phase I clinical test, ACAM2000 elicited significant epidermal responses, suppressing antibody and cell-induced reactions [85, 97]. Similarly, in the Phase II trial, double-blind, ACAM2000 has shown to have equal activity as Dryvax involving safety, antibody production, and cutaneous response level [85, 97]. In the Phase II vaccination trial, 100% of Dryvax and ACAM2000 vaccination participants had mild side effects; nevertheless, 50% of participants getting ACAM2000 suffered a fever, and their erythema was milder. Furthermore, no cases of increasing vaccinia or eczema have been reported in individuals who received ACAM2000 [85]. The rising incidences of myocarditis found in ACAM2000 and Dryvax vaccination participants were concerning [85]. According to the ACAM2000 vaccination trial, one in every 175 primary ACAM2000 vaccine participants developed myocarditis or pericarditis, which is comparable to Dryvax [85, 98]. Following a review, the U.S. FDA concluded that the long-term safety of ACAM2000 was not distinguishable from that of Dryvax, and ACAM2000 has been substituted with Dryvax [85, 98]. According to a WHO article (vaccines and immunization for Mpox), the ACAM2000 vaccine could be used for either pre- or postexposure prophylaxis against Mpox [99].

### 4.3. Alternative Therapies

Recently, researchers have been investigating alternative therapeutics for orthopoxviruses. Previously, a 20-mer EB peptide (NH2-RRKKAAVALLPAVLLALLAP-COOH) exhibited antiviral activity against viruses (orthopoxvirus, vaccinia, herpes simplex virus Type I, influenza) [100]. Altmann et al. reported that the peptide presented in vivo antiviral activity against vaccinia. It prevents viral invasion by blocking the attachment step [100]. Johnston and colleagues found that in vitro, interferon-*β* reduces Mpox viral replication and dissemination greatly in the human body [101]. IFN-*β* significantly blocked the MPXV when administered 6–8 h after infection, indicating its efficacy for application as a treatment [101]. The adoption of the RNAi (RNA interference) route as a novel method in antiviral therapeutic design is intriguing since viruses have small genomes with a restricted range of drug target genes [102]. Additionally, the variation of genetic elements between viral and mammalian genomes is beneficial in limiting off-target hits and potential adverse responses [102]. Nanoparticle interactions with macromolecules and pathogens are a growing subject of study [103]. Rogers and his coworkers reported in vitro that silver-based nanoparticles with diameters of about 10 nm (Ag-PS-10 and AgNO_3_) prevent MPXV infection [104].

### 4.4. Therapeutic Targets

The MPXV virus enters the body through broken skin, the respiratory system, or mucous membranes as part of its pathogenesis. Following entry, MPXV multiplies at the infection site before migrating to nearby lymph nodes and causing viremia. Numerous cell types are impacted by the virus, such as fibroblasts, macrophages, and epithelial cells [29, 38]. A new study found a collection of 49 genes conserved among the vertebrate and insect poxvirus groups [105]. Also, a total of 90 genes are conserved among chordate poxviruses and are necessary for poxvirus replication. Because these genes are greatly conserved and are probably essential for the replication of all orthopoxviruses, they are believed to feature several attractive targets for antiviral treatments [105]. The transcription process and its regulation require several proteins. These proteins, which are encoded by genes, are potential therapeutic targets. The D11L gene, which codes for a DNA-dependent ATPase, is one of them [106]. It is necessary for the premature deletion of transcripts from early transcriptional units' 3′ ends [105]. The A18R gene produces a crucial DNA helicase, and pathogens with mutations produce abnormally lengthy transcripts, implying that it is a key transcript releasing determinant [107, 108]. The main therapeutic target of Mpox identified by researchers are provided in Table 2.

## Figures and Tables

**Table 1:** Promising drugs for the treatment of monkeypox virus.

Drug	Trade/common name	IC50 value	Mechanism of action	Targeted protein/enzyme	Availability	Major side effects	Reference
Tecovirimat	Tpoxx	12.7 nM	Inhibits development of egress-competent orthopoxviruses	P37 protein	Oral	None reported	[55–57]
Cidofovir	Vistide	0.6 pM	Inhibits DNA polymerase by competing with dCTP	DNA polymerase	Injection	Fever, skin rash, nausea and vomiting, and nephrotoxicity	[55, 58–60]
Brincidofovir	Tembexa	2.76 nM	Prodrug that enhances bioavailability of cidofovir; inhibits viral DNA formation	Various viral DNA proteins	Oral	Mild nephrotoxicity	[55, 61, 62]
(N)-MCT	Nucleoside/nucleotide analogs	15 *μ*m	Inhibits viral DNA polymerases; may interfere with thymidylate synthetase	Viral DNA polymerases	N/A	None reported	[63, 64]
4′-thioIDU	Nucleoside/nucleotide analogs	6.3 *μ*M	Inhibits viral thymidine kinase	Viral thymidine kinase	N/A	None reported	[65, 66]
KAY-2-41	Nucleoside/nucleotide analogs	49 *μ*m	Shows antiviral activity against vaccinia, cowpox, and camelpox viruses	N/A	N/A	None reported	[67, 68]
NIOCH-14	Nucleoside/nucleotide analogs	—	Demonstrates potential as an antismallpox agent; suppresses virus quantity in critical organs	N/A	N/A	None reported	[69]
CMLDBU6128	Nucleoside/nucleotide analogs	—	Inhibits late gene expression and multiplication of poxviruses	Poxvirus gene expression	N/A	None reported	[70]

**Table 2:** Potential therapeutic targets of monkeypox virus.

Therapeutic target	Function and significance	Potential drugs	Reference
P37 envelope protein	A prospective target for antiviral drug design, P37 is essential for DNA replication	Tecovirimat	[56]
Thymidylate kinase	The monkeypox virus needs this protein to develop and mature, making it a potential treatment target	Sulopenem etzadroxil	[109]
DNA-dependent RNA polymerase subunit (A6R)	A6R is required for viral replication and is a possible therapeutic target for the monkeypox virus	Fludarabine	[110]
D8L protein	D8L is involved in cell entry and may be a therapeutic target for the monkeypox virus	—	[109]
F13L (major envelope protein)	F13L envelops intracellular adult viral particles and may be a potential therapy for the monkeypox virus	Tecovirimat	[111]
I7L (cysteine proteinase)	Due to its critical role in the replication of viruses by breaking down precursor polyproteins, the cysteine proteinase I7L is a good medicinal target	TTP-6171	[111, 112]

## Data Availability

All data were provided with references in the text and in the reference list.
